# Sleep Quality, Sleep Structure, and PER3 Genotype Mediate Chronotype Effects on Depressive Symptoms in Young Adults

**DOI:** 10.3389/fpsyg.2020.02028

**Published:** 2020-08-26

**Authors:** Chloe Weiss, Kerri Woods, Allan Filipowicz, Krista K. Ingram

**Affiliations:** ^1^Department of Biology, Colgate University, Hamilton, NY, United States; ^2^Samuel Curtis Johnson Graduate School of Management, Cornell University, Ithaca, NY, United States

**Keywords:** chronotype, sleep structure, depression, circadian mechanism, clock genes, PER3 variable number of tandom repeats, social jetlag

## Abstract

Depression and its related mood disorders are a major global health issue that disproportionately affects young adults. A number of factors that influence depressive symptoms are particularly relevant to the young adult developmental stage, including sleep loss, poor sleep quality, and the tendency toward eveningness in circadian preferences. However, relatively few studies have examined the relationship between sleep and circadian phenotypes, and their respective influences on mood, or considered potential molecular mechanisms driving these associations. Here, we use a multi-year, cross-sectional study of 806 primarily undergraduates to examine the relationships between sleep-wake chronotype, sleep disturbance, depression and genotypes associated with the PER3 variable number of tandom repeats (VNTR) polymorphism—circadian gene variants associated with both chronotype and sleep homeostatic drive. In addition, we use objective, Fitbit-generated sleep structure data on a subset of these participants (*n* = 67) to examine the relationships between chronotype, depression scores, actual measures of sleep duration, social jetlag, and the percent of deep and rapid eye movement (REM) sleep per night. In this population, chronotype is weakly associated with depressive symptoms and moderately correlated with self-reported sleep disturbance. Sleep disturbance is significantly associated with depression scores, but objective sleep parameters are not directly correlated with Beck Depression Inventory (BDI-II) scores, with the exceptions of a moderate correlation between social jetlag and depression scores in females and a marginal correlation between sleep duration and depression scores. Multiple regression and path analyses reveal that chronotype effects on depressive symptoms in this population are mediated largely by sleep disturbance. The PER3 VNTR genotype significantly predicts depressive symptoms in a model with objective sleep parameters, but it does not significantly predict depressive symptoms in a model with chronotype or subjective sleep disturbance. Interestingly, PER3^5,5^ genotypes, in males only, are independently related to chronotype and depression scores. Our results support hypotheses linking subjective sleep quality and chronotype and provide a first step in understanding how objective sleep structure may be linked to chronotype and depressive symptoms. Our results also suggest that circadian gene variants may show sex-specific effects linking sleep duration and sleep structure to depression.

## Introduction

Depression and related disorders, such as anxiety, affect nearly one-fifth of the global population and disproportionately affect young adults (Steel et al., 2014); studies of sleep patterns in depressed individuals highlight a robust relationship between sleep duration and depression, with extreme long and short sleep duration associated with increased depression (Watson et al., 2014; Zhai et al., 2015; Kalmbach et al., 2017). Individuals struggling with depression also report reduced sleep quality (Alvaro et al., 2013; Bakotic et al., 2017; Dinis and Bragança, 2018), and some studies have noted interactions between sleep quality and duration, suggesting more complex relationships between sleep variables and mood (Sandman et al., 2015; Bakotic et al., 2017; Kalmbach et al., 2017).

The two-process model of sleep regulation posits that sleep patterns are influenced by the physiological processes involved in sleep homeostasis and circadian rhythms (Kryger et al., 2010). Circadian rhythms regulate intrinsic daily cycles in physiological processes and behavior (Soria et al., 2010; McCarthy and Welsh, 2012; Zhang et al., 2016; Lazzerini Ospri et al., 2017) and are driven by intrinsic and extrinsic factors that control molecular oscillations in cells located in the suprachiasmatic nuclei (SCN). The molecular clockwork in the SCN coordinates a diverse set of widely dispersed clock mechanisms found in peripheral organs and cells *via* downstream activation and repression of circadian-controlled genes. Mutations in core regulatory clock genes are associated with both sleep and mood disorders, suggesting that molecular mechanisms affecting sleep and mood are influenced, in part, by the circadian clock (Randler, 2008; Mendlewicz, 2009; Soria et al., 2010; Lee et al., 2011; Courtet and Olié, 2012; McCarthy and Welsh, 2012; McClung, 2013; Robillard et al., 2013; Sivertsen et al., 2015; Antypa et al., 2016) and may utilize similar downstream pathways. Individuals vary in the timing of circadian sleep-wake patterns or chronotype (Adan et al., 2012; Antúnez et al., 2015); morning chronotypes (MT) wake up earlier in the morning and go to bed earlier than evening chronotypes (ET), with a majority of individuals showing intermediate patterns. The molecular basis for extreme chronotypes involves genetically-programmed advanced or delayed circadian phase (the timing of peak arousal driven by oscillations in clock genes) – extreme morning-types have advanced phases and extreme evening-types have delayed phases in oscillations relative to intermediate types, corresponding to their sleep-wake cycles (McClung, 2013; Robillard et al., 2013).

A leading hypothesis for how sleep-wake cycles is linked to depression involves “social jetlag,” a misalignment between socially induced activity patterns and endogenous sleep-wake cycles. Social jetlag is likely influenced by circadian misalignment, the degree to which the circadian phase is advanced or delayed relative to their socially-influenced sleep-wake cycle, particularly in individuals with extreme chronotypes. Studies on chronotypes or diurnal preference, the tendency towards “morningness” or “eveningness,” show that evening-types are more likely to experience symptoms of depression and/or anxiety (reviewed in Hidalgo et al., 2009; Kitamura et al., 2010; Levandovski et al., 2011; Adan et al., 2012; Merikanto et al., 2013, 2015; Prat and Adan, 2013; Fares et al., 2015; Antypa et al., 2016; Au and Reece, 2017; Van den Berg et al., 2018). Not surprisingly, a number of circadian clock DNA mutations are also associated with chronotype, sleep disturbance, and mood disorders (Archer et al., 2003; Partonen et al., 2007; Lavebratt et al., 2010a,b; Hida et al., 2014; Kim et al., 2015; Liu et al., 2015; Zhang et al., 2016).

The relationship between chronotype and sleep phenotype is less well-understood. Eveningness is associated with delayed sleep onset, as measured by both objective and subjective methods (Yadav and Singh, 2014; Van der Maren et al., 2018; Moderie et al., 2019). Evening orientation has also been associated with shorter sleep duration on work days and poorer sleep quality (Roepke and Duffy, 2010; Kim et al., 2012). Evening-types tend to self-report poor sleep quality (Fernández-Mendoza et al., 2010; Kitamura et al., 2010; Lemoine et al., 2013; Merikanto et al., 2013), and studies have shown that evening-types get less sleep on work days, increasing the sleep deficit by the weekend (Yadav et al., 2016); this sleep-related social jetlag is associated with depression (Hasler et al., 2010; Wittmann et al., 2010; Baum et al., 2014). Interestingly, studies incorporating the effects of both chronotype and sleep quality on depression have reported conflicting results. Van den Berg et al. (2018) found that chronotype effects on depressive symptoms were mediated strongly by poor sleep quality in evening-types in Dutch students (Van den Berg et al., 2018). These results are supported by earlier studies showing a relationship between chronotype, sleep quality or duration, and negative affect (Baum et al., 2014; Li et al., 2016), but sleep parameters did not fully mediate eveningness effects on depression in all studies (Kitamura et al., 2010; Simor et al., 2014). In adult Hungarians, Simor et al. (2014) reported preference for eveningness as an independent risk factor for depression, with insomniac symptoms acting as a partial mediator between diurnal preference and negative affect.

Overall, the results from these studies suggest that misalignment in circadian-related, sleep-wake patterns may increase negative affect in some populations, but due to the paucity of studies that explore mechanisms underlying both circadian misalignment and sleep, the molecular mechanisms underlying the links between sleep, circadian rhythms, and negative affect are unknown. One candidate mechanism is the PER3 gene, a highly rhythmic, circadian-related gene with expression in both the central nervous system and peripheral tissues (Archer et al., 2018). In humans, variants of the PER3 gene are associated with chronotype, sleep homeostasis, and mental disorders (Archer et al., 2003; Johansson et al., 2003; Akashi et al., 2010; McClung, 2013; Hida et al., 2014; Zhang et al., 2016; Liberman et al., 2017, 2018; Nguyen et al., 2019). A well-studied PER3 polymorphism in exon 18, rs57875989, encodes a variable number tandem repeat region (VNTR) containing a motif of 54 base pairs or 18 amino acids. This motif region encodes multiple CKI phosphorylation sites and the motif repeats either four or five times within the variant alleles. Genotypic variation in this PER3 polymorphism is associated with both diurnal preference and sleep phenotypes (Lázár et al., 2012). Morning-types are more likely to be homozygous for the 5-repeat allele (PER3^5/5^), and evening-types and individuals with delayed sleep phase disorder (DSPD) have greater frequencies of PER3^4/4^ homozygotes (Ebisawa et al., 2001; Archer et al., 2003, 2010; Pereira et al., 2005; Jones et al., 2007; Lázár et al., 2012; Liberman et al., 2017, 2018). For sleep phenotypes, PER3^5/5^ homozygotes show earlier sleep and wake times (Lázár et al., 2012) and multiple markers of greater homeostatic sleep pressure (reviewed in Dijk and Archer, 2010). Given the influence of the PER3 VNTR region on both chronotype and sleep homeostasis, this gene is a prime candidate for establishing molecular mechanisms connecting mood regulation to circadian and sleep homeostasis pathways.

In this study, we explore how chronotype and sleep parameters interact to influence depressive symptoms by measuring associations between parameters of all three behavioral phenotypes in a population of young adults. We incorporate objective measures of sleep structure [sleep duration, social jetlag, percentage of time spent in rapid eye movement (REM), and deep sleep] to better understand how features of sleep mediate chronotype effects on mood phenotypes. In addition, we test whether a circadian molecular marker, PER3 VNTR genotype, influences the effect of sleep quality and structure on depressive symptoms.

## Materials and Methods

### Participants

Data for the associations between chronotype, sleep disturbance, and depressive symptoms were collected from a multi-year study of a population of predominantly young adults [*n* = 806, 229 males, 577 females, median age: 19, range 17–36 (29 participants were faculty at the university; removal of these older individuals did not alter results, so they were included in the study)]. Participants were recruited primarily *via* an introductory biology course comprised of first or second-year undergraduates during the years 2013–2019; sampling occurred during the fall or spring, depending on the year. Participation rate for each sampling period ranged from 94 to 99%. Participants received laboratory credit and a food token for completing the survey study. A randomly selected subset of this study population provided the data used to measure sleep structure parameters (*n* = 67, 14 males, 53 females); these participants were compensated with a small monetary payment for wearing a Fitbit for a week. There was no difference in age, Patient-Reported Outcomes Measurement Information System (PROMIS), mid-sleep point on a free day (MSF), or Beck Depression Inventory (BDI-II) scores between the subset and the larger study population. Participants who did not have at least six nights of sleep data recorded, including two weekend nights, were excluded from the study. All methods adhered to the principles of the Declaration of Helsinki; the Institutional Review Board at Colgate University approved all procedures and consent forms (#FR-F13-07, #ER-F14-12, #F15-13, and #ER-F16-19). All participants gave written informed consent.

### Self-report Surveys

Participants participated in computer-based surveys which included the Munich Chronotype Questionnaire (MCTQ; Roenneberg et al., 2007), the PROMIS (Yu et al., 2011), and the BDI-II (Beck and Beamesderfer, 1974). The MCTQ is a self-reported measure of sleep-wake chronotype; MSF was used to designate the chronotype score, with the highest quartile MSF scores indicating eveningness (F:M ratio = 2.5:1) and the lowest quartile scores indicating morningness (F:M ratio = 5.8:1). Social jetlag was measured as MSF-MSW, the mid-sleep on work day. Because the measurement of circadian typology using MSF scores has some limitations, including weaker discrimination of morningness (Di Milia et al., 2013), we also measured diurnal preference using the Morningness-Eveningness Questionnaire (MEQ; Horne and Ostberg, 1976). Our analyses of relationships with MEQ scores, sleep factors, and depressive symptoms showed similar patterns to MSF chronotype results but were not statistically significant; thus, here, we report only the results for MSF scores. PROMIS is a self-reported measure of sleep disturbance, which ranges from 8 to 40. The score ranges correspond to sleep disturbance measures as follows: <25 non-slight, <29 mild, <37 moderate, and >38 severe. The BDI-II is a screening test for depression; diagnosis of depression combines scores on screening tests with clinical interviews. BDI-II scores are treated as quantized values and range from 0 to 60; individuals with scores <14 are not depressed, 14–19 are mildly depressed, 20–28 are moderately depressed, and >28 are severely depressed. The entire study was run over multiple years, and particular surveys were not used every year; thus, sample sizes differ for each analysis.

### Genotyping

Ten to twenty hair follicles were collected from each participant in order to determine genotypes for PER3 variable number of tandem repeats (VNTR; rs57875989). Hair samples were digested at 56°C for 24 h, and were then purified using the Qiagen DNAeasy Micro Kit. To measure the VNTR length polymorphism of 54 base pairs in exon 18 of the PER3 gene, we used a fragment length analysis on an ABI 3100 sequencer. The following PCR primers were used with the forward primer fluorescently labeled with 6-FAM: forward, 5'-CAAAATTTTATGACACTACCAGAATGGCTGAC-3', and reverse, 5'-AACCTTGTACTTCCACATCAGTGCCTGG-3' (Ebisawa et al., 2001). The PCR was performed in a 25-μl volume using Qiagen PCR Mastermix. The PCR cycling conditions were 3 min at 94°C, followed by 35 cycles of 45 s at 94°C, 45 s at 58°C, and 45 s at 72°C, with a final step at 72°C for 3 min. PER3 alleles were separated by capillary electrophoresis on an ABI 3700 sequencer and sized using ABI ROX standards. Participants were identified as PER3^4/4^, PER3^4/5^, or PER3^5/5^.

### FitBit Sleep Data

A subset of participants wore Fitbit® Charge 2 or Versa 2 personal activity monitors for 1 week. Fitbit devices use accelerometer-based measures to estimate sleep quality and sleep structure parameters and have been validated against clinical sleep monitors for comparable results for sleep onset and offset, sleep duration and efficiency, and sleep structure (de Zambotti et al., 2018; Liang and Martell, 2018). Our study focused on three sleep parameters: sleep duration (total minutes asleep per night), the percentage of deep sleep per night, and the percentage of REM sleep per night.

### Statistical Analysis

To investigate the relationships among the study variables, bivariate correlations were computed for Pearson’s coefficients. Multiple linear regressions (variables or variable sets entered sequentially) were used to test for relationships between BDI-II scores and MSF (chronotype) scores, BDI-II scores and sleep disturbance scores, and a combined analysis of BDI-II scores with chronotype and sleep disturbance scores. The inclusion of our subjective jetlag measure in regression models was problematic due to issues with collinearity tolerance (0.5); thus, we excluded it from the analyses in the first studies on chronotype and sleep disturbance. We used a multiple linear regression to test for relationships between BDI-II scores and objective sleep parameters (sleep duration, percent time in light, deep, and REM sleep, and social jetlag). Odds ratio tests were used to test differences between genotypic frequencies and chronotypes; we tested the hypotheses that frequencies of PER3^4,4^ homozygotes were higher in evening-types versus other genotypes (PER3^4,5^ and PER3^5,5^ combined), overall and separately for males and females. One-way analyses of variance (ANOVAs) were used to test for overall and sex-specific differences in MSF, BDI-II, and sleep disturbance scores and in sleep parameters in the sleep subset of data. We performed mediation analyses using the PROCESS macro (Hayes, 2009, 2013) that tested the role of sleep disturbance as a mediator of the relationship between chronotype and depressive symptoms. A bootstrapping procedure (with 5,000 bootstrap samples) was used; a 95% CI that does not include zero provides evidence of a significant indirect effect (Preacher and Hayes, 2008). For estimates of effect sizes for indirect effect, Preacher and Kelley (2011) suggested the use of standardized indirect effect. We used this convention to estimate the indirect effect of chronotype. All statistical analyses were performed in SPSS.

## Results

### Associations of Chronotype With Depressive Symptoms

**Figure 1:**
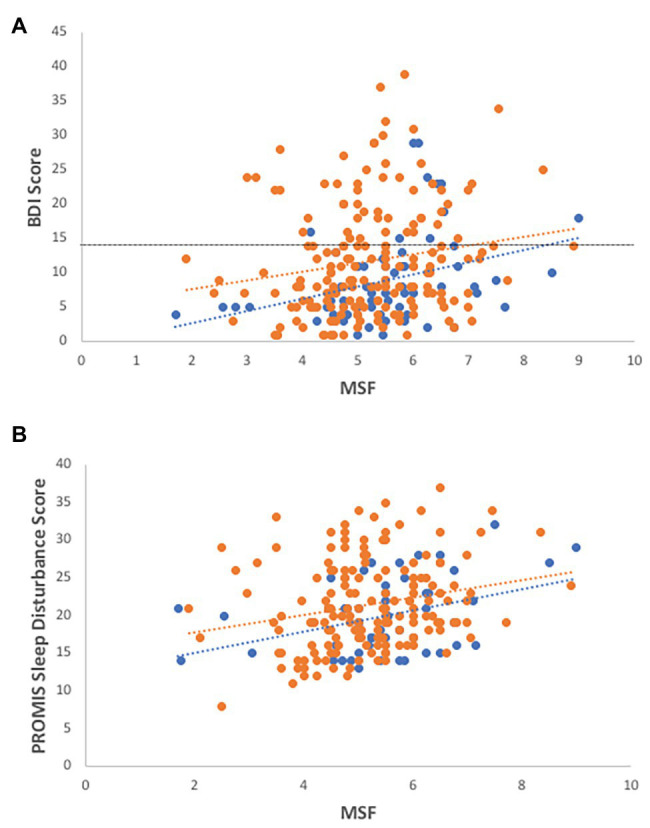
Associations of chronotype with depression and sleep disturbance. Evening-types demonstrate higher levels of **(A)** depression (*R*^2^ = 0.07, *F* = 3.39, *df* = 223, *p* = 0.002; *R^2^_males_* = 0.12, *R^2^_females_* = 0.03) and **(B)** sleep disturbance (*R*^2^ = 0.145, *F* = 5.163, *df* = 51, *p* = 0.029; *R^2^_males_* = 0.15, *R^2^_females_* = 0.05) than morning-types and intermediates, as measured by self-reported health surveys. Females-open circles, males-closed circles, and dotted line indicate cut-off for depressive symptoms using the Beck Depression Inventory (BDI-II) instrument.

In the overall dataset, MSF chronotype scores are weakly correlated with BDI-II depression scores (Figure 1A; *R*^2^ = 0.07, *F* = 3.39, *df* = 223, *p* = 0.002) with evening-types reporting greater symptoms of depression. Although the coefficient of MSF is significant, the addition of MSF scores to the model leads to a small change on the predictive power (Table 1).

**Table 1:** Association of mid-sleep point on a free day (MSF) chronotype with depressive symptoms.

Model	Variables entered	*β*	*r*	*f*^2^	*F*	*df*	*p*
1	Gender Age	0.16^*^ −0.13	0.21	0.04	4.93	223	0.008
2	Gender Age VNTR genotype	0.16^*^ −0.13 −0.05	0.21	0.02	3.28	222	0.022
3	Gender Age VNTR genotype MSF chronotype	0.19^**^ −0.07 −0.05 0.19^**^	0.27	0.03	4.39	221	0.002

*β* represents the standardized coefficient, Beta, for each variable entered; *f*^2^ represents the effect size change of model due to variables entered in a multiple linear regression.

* Represents *p* < 0.05.

** Represents *p* < 0.01.

In the sleep parameter subset, MSF scores were also correlated with BDI-II scores (*R*^2^ = 0.134, *F* = 7.41, *df* = 49, *p* = 0.009), with male ETs, in particular, reporting a positive association with depression scores (*R*^2^ = 0.266, *F* = 5.143, *df* = 11, *p* = 0.047).

### Associations of Chronotype With Sleep Variables

MSF scores are positively correlated with sleep disturbance scores as measured by self-reported PROMIS surveys: evening-types report more sleep disturbance than intermediate or morning-types (Figure 1B; *R*^2^ = 0.145, *F* = 5.163, *df* = 51, *p* = 0.029).

In the subset of objective sleep data, chronotype scores are not significantly correlated with percent age of time spent in REM (*R*^2^ = 0.079, *F* = 1.43, *df* = 51, *p* = 0.240), percentage of deep sleep (*R*^2^ = 0.002, *F* = 0.056, *df* = 38, *p* = 0.814), or sleep duration (*R*^2^ = 0.065, *F* = 2.782, *df* = 41, *p* = 0.103) but are strongly correlated with social jetlag (*R*^2^ = 0.439, *F* = 39.20, *df* = 51, *p* < 0.001), with evening-types experiencing more social jetlag.

### Associations of Sleep Parameters With Depressive Symptoms

**Figure 2:**
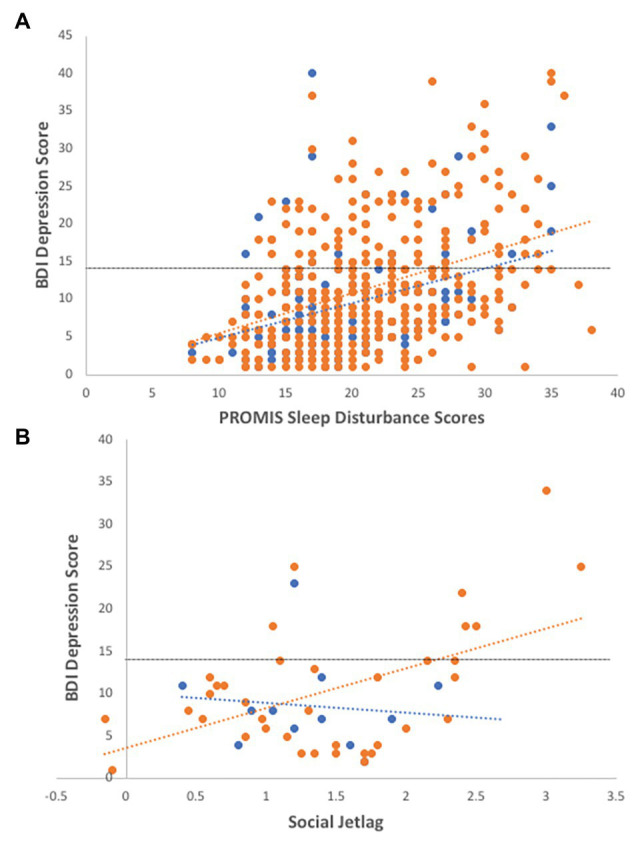
Associations of sleep disturbance and social jetlag with depression. **(A)** Individuals reporting higher sleep disturbance are more depressed, controlling for age and gender (*R*^2^ = 0.14, *F* = 4.53, *df* = 429, *p* = 0.011; *R^2^_males_* = 0.13, *R^2^_females_* = 0.17). **(B)** In the sleep subset of data, social jetlag is significantly correlated with BDI-II depression scores (*R*^2^ = 0.13, *df* = 49, *p* = 0.009), particularly in females (*R*^2^ = 0.26, *F* = 7.80, *df* = 37, *p* = 0.008). Females-open circles, males-closed circles, and dotted line indicate cut-off for depressive symptoms using the BDI-II instrument.

Overall, self-reported sleep disturbance scores are significantly associated with depression scores (Figure 2A; *R*^2^ = 0.161, *F* = 20.558, *df* = 431, *p* < 0.001). In a model with gender, age, and genotype, the addition of sleep disturbance scores significantly increases the prediction of depressive symptoms with a medium effect size (Table 2).

**Table 2:** Association of sleep disturbance with depressive symptoms.

Model	Variables entered	*β*	*r*	*f*^2^	*F*	*df*	*p*
1	Gender Age	0.12^*^ −0.07	0.14	0.02	4.53	429	0.011
2	Gender Age VNTR genotype	0.12^*^ −0.08 −0.007	0.15	0.02	3.11	428	0.026
3	Gender Age VNTR genotype Sleep disturbance	0.10^*^ −0.05 −0.005 0.38^**^	0.40	0.16	20.56	427	<0.001

*β* represents the standardized coefficient, Beta, for each variable entered; *f*^2^ represents the effect size change of model due to variables entered in a multiple linear regression.

* Represents *p* < 0.05.

** Represents *p* < 0.01.

In the subset of sleep data, depression scores were correlated with social jetlag (Figure 2B; *R*^2^ = 0.13, *df* = 49, *p* = 0.009), particularly in females (*R*^2^ = 0.26, *F* = 7.80, *df* = 37, *p* = 0.008), and marginally correlated with sleep duration (*R*^2^ = 0.10, *F* = 5.562, *df* = 52, *p* = 0.022), with depressed females showing longer sleep durations (*R*^2^ = 0.17, *df* = 37, *p* = 0.03). Social jetlag was associated with sleep duration (*R*^2^ = 0.145, *F* = 6.94, *df* = 42, *p* = 0.012) but not sleep disturbance or other sleep variables.

### Mediation of Chronotype Effects on Depressive Symptoms by Sleep Factors

A multiple regression combining the effects of chronotype and sleep disturbance on depressive symptoms reveals that the significant effects of chronotype become non-significant on addition of sleep disturbance scores in the model (Table 3). The model effect size shows a moderate increase with the addition of sleep disturbance (*f*^2^ = 0.11), and gender and sleep disturbance scores have significant Beta values (*β* = 0.15 and 0.32, respectively). In a mediation path analysis, MSF chronotype has a significant total effect on depression score [*c_t_* = 1.16 (0.51); *p* < 0.05], but this effect is strongly mediated by sleep disturbance [Figure 4; indirect effect of *SD* = 0.41 (CI: 0.07, 0.84)].

**Table 3:** Combined associations of MSF chronotype and sleep disturbance with depressive symptoms.

Model	Variables entered	*β*	*r*	*f*^2^	*F*	*df*	*p*
1	Gender Age	0.17^*^ −0.11	0.21	0.04	3.79	174	0.024
2	Gender Age VNTR genotype	0.17^*^ −0.11 0.02	0.21	0.006	2.88	173	0.038
3	Gender Age VNTR genotype MSF chronotype	0.19^*^ −0.12 0.02 0.17^*^	0.27	0.031	3.48	172	0.009
4	Gender Age VNTR genotype MSF chronotype Sleep disturbance	0.15^*^ −0.15^*^ 0.02 0.09 0.32^***^	0.41	0.11	7.03	171	<0.001

*β* represents the standardized coefficient, Beta, for each variable entered; *f*^2^ represents the effect size change of model due to variables entered in a multiple linear regression.

* Represents *p* < 0.05.

** Represents *p* < 0.01.

*** Represents *p* < 0.001.

### Associations of PER3 VNTR Genotype With Chronotype, Sleep, and Depression Scores

**Figure 3:**
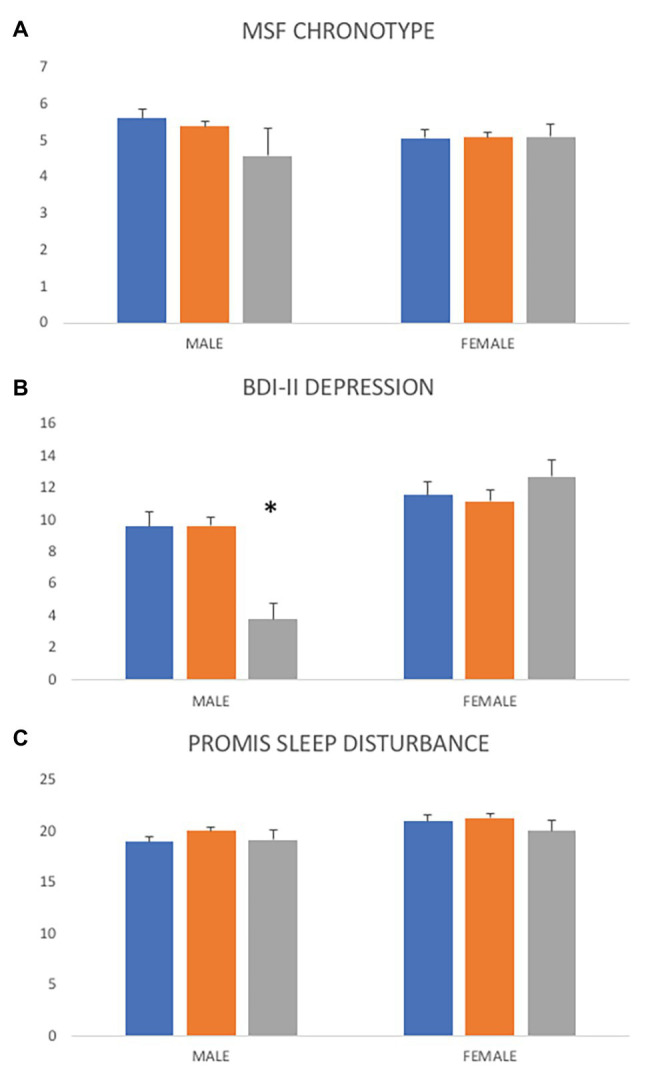
Differences in chronotype, depression, and sleep disturbance scores across PER3 variable number of tandom repeats (VNTR) genotypes. **(A)** Overall, PER3 VNTR genotypes are not significantly associated with average chronotype scores (*F* = 0.31, *df* = 266, *p* = 0.737) but ET males are nine times more likely to have a PER3^4,4^ genotype (OR = 9.37, CI: 1.12–78.17, *z* = 2.07, *p* = 0.038). **(B)** Overall average depression scores do not vary across PER3 VNTR genotypes (*F* = 0.07, *df* = 540, *p* = 0.929) but males with a PER3^5,5^ genotype tend to have lower depressions scores than males of other genotypes (*F* = 2.09, *df* = 129, *p* = 0.064). **(C)** Average sleep disturbance scores are not significantly different across PER3 VNTR genotypes (*F* = 1.45, *df* = 865, *p* = 0.236). PER3^4,4^ = blue, PER3^4,5^ = orange, PER3^5,5^ = gray. ^*^p < 0.05.

PER3 VNTR genotypes do not differ significantly across overall average MSF (Figure 3; *F*_2,266_ = 3.1, *p* = 0.737), sleep disturbance (*F*_2,865_ = 1.45, *p* = 0.236), or depression scores (*F*_2,540_ = 0.07, *p* = 0.929). In the larger data set, VNTR genotypes are not associated with chronotype categories; PER3^4,4^ genotypes are not more likely to be evening-types (OR = 0.851, CI: 0.38–1.76, *z* = 0.51, *p* = 0.612). However, ET males are nine times more likely to have a PER3^4,4^ genotype (Figure 3; OR = 9.37, CI: 1.12–78.17, *z* = 2.07, *p* = 0.038) than MT males. In addition, males with a PER3^5,5^ genotype tend to have lower depression scores than males of other genotypes, but this result was not significant (*F* = 2.09, *df* = 129, *p* = 0.064). Overall, VNTR genotypes have marginal effects in predicting depressive symptoms in regressions of MSF chronotype and sleep disturbance (Tables 1 and 2).

**Figure 4:**
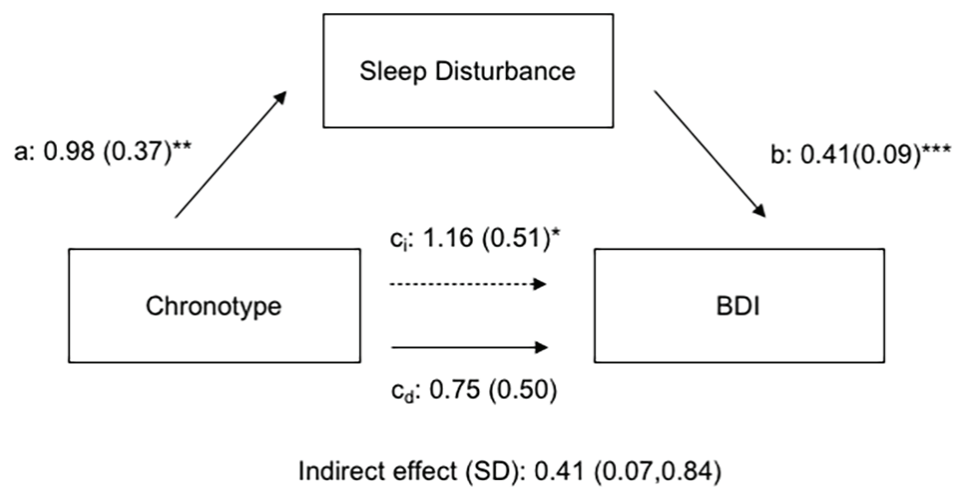
Path coefficients for mediation analysis of chronotype effects on depression by self-reported sleep disturbance. Effects of chronotype on depression are mediated by indirect effects of sleep disturbance. a, b, c_i_, and c_d_ are regression coefficients (ET have higher chronotype scores; i subscript indicates indirect effect; and d subscript indicates direct effect). ^*^p < 0.05, ^**^p < 0.01, and ^***^p < 0.001.

In an exploratory regression analysis on the subset of sleep data, objective sleep parameters (percentage of REM, deep sleep, sleep duration, and social jetlag) are not significant predictors of depressive symptoms (Table 4). However, gender and VNTR genotype are strong predictors (*β* = 0.36 and −0.44, respectively).

**Table 4:** Associations of PER3 VNTR genotypes and Fitbit®-measured sleep parameters with depressive symptoms.

Model	Variables entered	*β*	*r*	*f*^2^	*F*	*df*	*p*
1	Gender Percentage of REM Percentage of deep Duration Jetlag VNTR	0.36^*^ −0.26 0.17 0.05 0.28 −0.44^**^	0.63	1.7	3.16	38	0.014

*β* represents the standardized coefficient, Beta, for each variable entered; *f*^2^ represents the effect size change of model due to variables entered in a multiple linear regression.

* Represents *p* < 0.05.

** Represents *p* < 0.01.

## Discussion

Overall, our study reveals complex and, potentially, sex-specific relationships between depressive symptoms and chronotype, sleep disturbance, and sleep structure. Our results confirmed previous reports that chronotype is weakly associated with depression scores (Hidalgo et al., 2009; Kitamura et al., 2010; Merikanto et al., 2013, 2015; Antypa et al., 2016; Au and Reece, 2017; Van den Berg et al., 2018), with evening-types having higher BDI-II scores. Social jetlag values were highly correlated to MSF and depression scores; ET individuals experience greater social jetlag and individuals experiencing greater jetlag reported more depressive symptoms, particularly in females. Our results parallel findings reported by Levandovski et al. (2011) for a rural population of middle-aged adults but contradict results of clinical study of patients with MDD by Knapen et al. (2018) in which the authors found no direct association between depression scores and social jetlag in MDD or control groups (Levandovski et al., 2011; Knapen et al., 2018). However, in our young adult population, factors involved in sleep quality and structure are more strongly associated with depressive symptoms than chronotype or circadian misalignment. Self-reported sleep disturbance scores explained nearly 20% of the overall variance in BDI-II scores and over 30% of the variance in male BDI-II scores. In addition, when controlling for gender and genotype, nearly 40% of the variance in depression scores can be explained by objective measures of sleep structure, including the percentage of time spent in REM sleep, deep sleep, sleep duration, and social jetlag, indicating a potential for an interaction of both circadian (chronotype) and sleep homeostatic (sleep structure and quality) influences on depressive symptoms.

Interestingly, chronotype was significantly associated with overall self-reported sleep disturbance scores, with evening-types reporting higher sleep disturbance, but not with objective sleep structure parameters or sleep duration. In addition, the degree of misalignment of circadian rhythms with the social environment, or social jetlag, was associated with only one sleep parameter, sleep duration; individuals with greater social jetlag slept longer on free days. Our results on the influence of eveningness on self-reported sleep disturbance support previous studies showing subjective sleep quality measures are associated with chronotype (Kitamura et al., 2010; Roeser et al., 2013; Rique et al., 2014; Yun et al., 2015). For objective sleep structure, we did not find significant associations between sleep structure and chronotype. Previous research has shown eveningness to be related to shorter sleep duration (Park et al., 1998; Soehner et al., 2011); we see this trend in our sleep structure data, but the trend is not significant within our sample of young adults. It is possible that differences in the “quality” of the sleep structure, rather than overall duration of each sleep type, as measured in our Fitbit study, are the relevant differences to consider for chronotype effects on depression. In previous studies, evening-types have been shown to have differences in EEG or slow wave activity during REM (Viola et al., 2007) and non-REM (Mongrain et al., 2006; Viola et al., 2007) sleep.

Our study does provide another potential factor to consider – the gender effect seen in our Fitbit-generated sleep structure data. We find that males have significant relationships between chronotype and sleep variables, with evening-types having significantly greater percentage of REM sleep and a lower percentage of deep sleep. However, the variance in these sleep factors across chronotype is greater in females. If sleep structure is related to depression, one might predict an increase in REM sleep and a decrease in restorative deep sleep in individuals battling with mood disorders (Palagini et al., 2013). Although the percentage of time in REM or deep sleep for males did not directly predict BDI-II scores, our sample size for sleep structure in males is small, and we did not have many males reporting depressive symptoms. We also see significant differences between genders in percentage of REM sleep and self-reported sleep disturbance, with females reporting more disturbance and experiencing more REM sleep per night than males. These results are in line with the higher incidence of depression in females, particularly in young adults. Although males and females did not significantly differ in levels of social jetlag, females showed a significant positive relationship between social jetlag and depression scores, similar to the study of Regestein et al. (2010), which reported a higher risk of melancholic symptoms in females with a sleep debt of at least 2 h (Regestein et al., 2010). These results suggest that the relative risk for sleep-related depressive symptoms may be higher for females, particularly those experiencing circadian misalignment.

Our path analysis reveals that the effects of chronotype on depressive symptoms are largely mediated *via* effects on subjective sleep disturbance. These results support findings from previous studies in young adults (Kitamura et al., 2010; Bakotic et al., 2017; Van den Berg et al., 2018), suggesting that depressive symptoms in evening-type young adults may be primarily a consequence of their poor sleep quality. However, this interaction between sleep and chronotype may not extend to other populations or older adults, where chronotype plays a small, but independent, role in depression risk (Giannotti et al., 2002; Simor et al., 2014). Measures of self-reported sleep quality mask multiple variables, including sleep duration, sleep architecture, and sleep structure (percentage of time spent in light, deep, and REM sleep and the number awakenings during sleep). Thus, it is difficult to discern from sleep quality studies which factors to target for potential translational therapies. Because most sleep studies to date rely on self-report surveys, it is important to validate the critical sleep factors that influence negative affect using more objective measures of sleep such as the percentage of time spent in deep sleep or REM; such information can be gleaned from sleep monitors (de Zambotti et al., 2018; Liang and Martell, 2018). Given the limited sample size for our objective sleep structure parameters, our results suggest that more extensive studies should explore how factors involved in sleep structure mediate chronotype effects on depression, as improving quality and duration of sleep may be more efficient therapies than modifying sleep-wake patterns.

Few studies have addressed mechanisms for how circadian and sleep homeostatic processes might be connected, but limited studies have focused primarily on the PER3 VNTR polymorphism (Archer et al., 2003, 2010; Dallaspezia et al., 2011; Lázár et al., 2012; Hida et al., 2014; Turco et al., 2017). With a relatively large sample, we found that VNTR variants in this population are not significantly associated with MSF, sleep disturbance, or depression scores. Previously, Lázár et al. (2012) found a significant association with both diurnal preference and sleep-wake timing in a sample comprised of greater than 60% males; the current study is comprised of only 28% males. Interestingly, if we split the data set by gender, we find a significant gender effect in the association between the PER3 VNTR polymorphism and MSF chronotype with male PER3^4,4^ genotypes nine times more likely to be evening-types than other genotypes. The results from these two studies suggest that there may be a gender effect in the association between the PER3 VNTR polymorphism and MSF chronotype; such sex-dependent effects have been found for other circadian-related genes, including a sex-specific association of a PER3 single nucleotide polymorphism (SNP rs228697) with depressive symptoms (Shi et al., 2016).

A new aspect of this study was the exploration of the association of the PER3 VNTR with objectively-measured sleep variables. A number of studies have reported associations between PER3 VNTR genotype and sleep duration or timing (Archer et al., 2003, 2010; Lázár et al., 2012; Hida et al., 2014; Turco et al., 2017) and sleep structure (Viola et al., 2007, 2011; Goel et al., 2009), supporting the hypothesis that PER3 acts to help regulated sleep homeostasis. In our smaller sleep study, the VNTR polymorphism did not directly correlate with measured sleep parameters, as expected due to our limited sample size for genetic associations. However, we did find that the addition of VNTR genotype added significant explanatory power to the association between measures of sleep structure (percentage of REM, sleep duration, and, to a lesser extent, deep sleep) and depressive symptoms, suggesting there may be an underlying shared molecular pathway regulating circadian rhythms and sleep structure that influences mood pathways and involves the PER3 gene. Such a link was proposed by Dijk and Archer (2010) and by Lázár et al. (2012) but has yet to be supported by experimental data (Dijk and Archer 2010; Lázár et al., 2012).

The strengths of our study include a large sample size for measures of chronotype, sleep disturbance, and depression scores, the addition of social jetlag measures—a factor in sleep-wake regulation that is understudied in epidemiological studies (Wittmann et al., 2006), and the inclusion of objective sleep structure measures using Fitbit actigraphy. Our study is limited by our cross-sectional design which does not allow us to infer directional or causal relationships between our circadian and sleep variables and negative affect. In addition, we did not include an independent analysis of subjective wakefulness in our study of objective sleep parameters and subjective sleep disturbance (Adan, 1993). Our use of the BDI-II to measure depressive symptoms also has limitations in elucidating effects of sleep factors, as some of the questions involve sleep-related issues; thus, there may be partial autocorrelation between sleep disturbance measures and BDI-II measures. Other limitations include the relatively small sample size of males and the restricted age range in our sleep structure subset of data. Young adults tend to be more ETs and this may dampen effects of chronotype on negative affect. In addition, our sleep structure subset consisted of primarily Caucasian undergraduates, a population that is more likely to exhibit high sleep disturbance and low average sleep duration; thus, our results may not extend to the general population or populations of non-Caucasian descent. It is also possible that a study of depressive symptoms in undergraduates may be affected by other common factors that we did not account for (i.e., academic stress and alcohol use).

The high prevalence of mood and sleep disorders is a national and global concern – a public health issue with major economic and social costs. Young adults undergo a developmental transition in their chronobiology that affects the timing of their circadian rhythms and may be at higher risk for circadian‐ and sleep-related depression. The combination of later sleep-wake cycles and poor sleep habits may contribute to the high rates of depression reported in this age group. Here, we show that both chronotype and sleep quality/structure are associated with depressive symptoms and that PER3 genotype might mediate this association. Our results suggest interesting implications for how the knowledge of circadian and sleep phenotypes may be applied to therapeutic efforts. First, gender may play a role in whether an individual’s circadian or sleep phenotype may be a risk factor for depression. Second, an individual’s circadian genotype might provide clues to how disruption in sleep patterns may confer a risk of depression. Finally, although age was not rigorously tested in these current studies, the fact that chronotype tends to more strongly predict depressive symptoms in older populations, while sleep factors seem to predominate in populations of young adults, suggests that age might be an important factor in assessing circadian and sleep influences on depression. Understanding how chronotype and sleep interact to affect depressive symptoms may yield highly effective, non-invasive behavioral remedies (minimum sleep recommendations, blue light therapy, “time-out” for screen-time, etc.) that improve the quality of life and success of undergraduate students and other vulnerable populations.

## Data Availability Statement

The raw data supporting the conclusions of this article will be made available by the authors, without undue reservation.

## Ethics Statement

The studies involving human participants were reviewed and approved by Institutional Review Boards of Colgate and Cornell Universities. The patients/participants provided their written informed consent to participate in this study.

## Author Contributions

KI designed the study. KW organized the study and database. KI, CW, and AF performed the statistical analysis. KI wrote the draft of the manuscript. All authors contributed to manuscript revision and approved the submitted version.

### Conflict of Interest

The authors declare that the research was conducted in the absence of any commercial or financial relationships that could be construed as a potential conflict of interest.

## References

[ref1] Adan A. (1993). Circadian variations in psychological measures: a new classification. Chronobiologia 20, 145–161. PMID: 8131664

[ref2] Adan A. Archer S. N. Hidalgo M. P. Di Milia L. Natale V. Randler C. (2012). Circadian typology: a comprehensive review. Chronobiol. Int. 29, 1153–1175. 10.3109/07420528.2012.719971, PMID: 23004349

[ref3] Akashi M. Soma H. Yamamoto T. Tsugitomi A. Yamashita S. Yamamoto T.. (2010). Noninvasive method for assessing the human circadian clock using hair follicle cells. Proc. Natl. Acad. Sci. U. S. A. 107, 15643–15648. 10.1073/pnas.1003878107, PMID: 20798039 PMC2932591

[ref4] Alvaro P. K. Roberts R. M. Harris J. K. (2013). A systematic review assessing bidirectionality between sleep disturbances, anxiety, and depression. Sleep 36, 1059–1068. 10.5665/sleep.2810, PMID: 23814343 PMC3669059

[ref5] Antúnez J. M. Navarro J. F. Adan A. (2015). Circadian typology is related to resilience and optimism in healthy adults. Chronobiol. Int. 32, 524–530. 10.3109/07420528.2013.790397, PMID: 25799223

[ref6] Antypa N. Vogelzangs N. Meesters Y. Schoevers R. Penninx B. W. (2016). Chronotype associations with depression and anxiety disorders in a large cohort study. Depress. Anxiety 33, 75–83. 10.1002/da.22422, PMID: 26367018

[ref7] Archer S. N. Carpen J. D. Gibson M. Lim G. H. Johnston J. D. Skene D. J.. (2010). Polymorphism in the PER3 promoter associates with diurnal preference and delayed sleep phase disorder. Sleep 33, 695–701. 10.1093/sleep/33.5.695, PMID: 20469812 PMC2864885

[ref8] Archer S. N. Robilliard D. L. Skene D. J. Smits M. Williams A. Arendt J.. (2003). A length polymorphism in the circadian clock gene Per3 is linked to delayed sleep phase syndrome and extreme diurnal preference. Sleep 26, 413–415. 10.1093/sleep/26.4.413, PMID: 12841365

[ref91] Archer S. N. Schmidt C. Vandewalle G. Dijk D. J. (2018). Phenotyping of PER3 variants reveals widespread effects on circadian preference, sleep regulation, and health. Sleep Med. Rev. 40, 109–126. 10.1016/j.smrv.2017.10.008 29248294

[ref9] Au J. Reece J. (2017). The relationship between chronotype and depressive symptoms: a meta-analysis. J. Affect. Disord. 218, 93–104. 10.1016/j.jad.2017.04.021, PMID: 28463712

[ref10] Bakotic M. Radosevic-Vidacek B. Bjelajac A. K. (2017). Morningness-eveningness and daytime functioning in university students: the mediating role of sleep characteristics. J. Sleep Res. 26, 210–218. 10.1111/jsr.12467, PMID: 27758010

[ref11] Baum K. T. Desai A. Field J. Miller L. E. Rausch J. Beebe D. W. (2014). Sleep restriction worsens mood and emotion regulation in adolescents. J. Child Psychol. Psychiatry 55, 180–190. 10.1111/jcpp.12125, PMID: 24889207 PMC4047523

[ref12] Beck A. T. Beamesderfer A. (1974). “Assessment of depression: the depression inventory” in Psychological measurements in psychopharmacology. eds. Pichot P. Olivier-Martin R. (Basel, Switzerland: Karger), 151–169.

[ref14] Courtet P. Olié E. (2012). Circadian dimension and severity of depression. Eur. Neuropsychopharmacol. 22(Suppl. 3), S476–S481. 10.1016/j.euroneuro.2012.07.009, PMID: 22959112

[ref15] Dallaspezia S. Lorenzi C. Pirovano A. Colombo C. Smeraldi E. Benedetti F. (2011). Circadian clock gene Per3 variants influence the postpartum onset of bipolar disorder. Eur. Psychiatry 26, 138–140. 10.1016/j.eurpsy.2010.11.009, PMID: 21316201

[ref16] de Zambotti M. Goldstone A. Claudatos S. Colrain I. M. Baker F. C. (2018). A validation study of Fitbit Charge 2™ compared with polysomnography in adults. Chronobiol. Int. 35, 465–476. 10.1080/07420528.2017.1413578, PMID: 29235907

[ref17] Di Milia L. Adan A. Vicenzo N. Randler C. (2013). Reviewing the psychometric properties of contemporary circadian typology measures. Chronobiol. Int. 30, 1261–1271. 10.3109/07420528.2013.817415, PMID: 24001393

[ref18] Dijk D. J. Archer S. N. (2010). PERIOD3, circadian phenotypes, and sleep homeostasis. Sleep Med. Rev. 14, 151–160. 10.1016/j.smrv.2009.07.002, PMID: 19716732

[ref19] Dinis J. Bragança M. (2018). Quality of sleep and depression in college students: a systematic review. Sleep Sci 11, 290–301. 10.5935/1984-0063.20180045, PMID: 30746048 PMC6361309

[ref20] Ebisawa T. Uchiyama M. Kajimura N. Mishima K. Kamei Y. Katoh M.. (2001). Association of structural polymorphisms in the human period3 gene with delayed sleep phase syndrome. EMBO Rep. 2, 342–346. 10.1093/embo-reports/kve070, PMID: 11306557 PMC1083867

[ref21] Fares S. Hermens D. F. Naismith S. L. White D. Hickie I. B. Robillard R. (2015). Clinical correlates of chronotypes in young persons with mental disorders. Chronobiol. Int. 32, 1183–1191. 10.3109/07420528.2015.1078346, PMID: 26375049

[ref22] Fernández-Mendoza J. Ilioudi C. Montes M. I. Olavarrieta-Bernardino S. Aguirre-Berrocal A. De La Cruz-Troca J. J. (2010). Circadian preference, nighttime sleep and daytime functioning in young adulthood. Sleep Biol. Rhythms 8, 52–62. 10.1111/j.1479-8425.2010.00430.x

[ref23] Giannotti F. Cortesi F. Sebastiani T. Ottaviano S. (2002). Circadian preference, sleep and daytime behaviour in adolescence. J. Sleep Res. 11, 191–199. 10.1046/j.1365-2869.2002.00302.x, PMID: 12220314

[ref24] Goel N. Bank S. Mignot E. Dinges D. F. (2009). PER3 polymorphism predicts cumulative sleep homeostatic but not neurobehavioral changes to chronic partial sleep deprivation. PLoS One 4:e5874. 10.1371/journal.pone.0005874, PMID: 19516903 PMC2689932

[ref25] Hasler B. P. Buysse D. J. Kupfer D. J. Germain A. (2010). Phase relationships between core body temperature, melatonin, and sleep are associated with depression severity: further evidence for circadian misalignment in non-seasonal depression. Psychiatry Res. 178, 205–207. 10.1016/j.psychres.2010.04.027, PMID: 20471106 PMC2914120

[ref26] Hayes A. F. (2009). Beyond Baron and Kenny: statistical mediation analysis in the new millennium. Commun. Monogr. 76, 408–420. 10.1080/03637750903310360

[ref27] Hayes A. F. (2013). Introduction to mediation, moderation, and conditional process analysis: A regression-based approach. New York, NY: Guilford publications.

[ref28] Hida A. Kitamura S. Katayose Y. Kato M. Ono H. Kadotani H.. (2014). Screening of clock gene polymorphisms demonstrates association of a PER3 polymorphism with morningness-eveningness preference and circadian rhythm sleep disorder. Sci. Rep. 4, 1–6. 10.1038/srep06309, PMID: 25201053 PMC4158573

[ref29] Hidalgo M. P. Caumo W. Posser M. Coccaro S. B. Camozzato A. L. Chaves M. L. F. (2009). Relationship between depressive mood and chronotype in healthy subjects. Psychiatry Clin. Neurosci. 63, 283–290. 10.1111/j.1440-1819.2009.01965.x, PMID: 19566758

[ref30] Horne J. A. Ostberg O. (1976). A self-assessment questionnaire to determine morningness-eveningness in human circadian rhythms. Int. J. Chronobiol. 4, 97–110. PMID: 1027738

[ref13] Johansson C. Willeit M. Smedh C. Ekholm J. Paunio T. Kieseppä T.. (2003). Circadian clock-related polymorphisms in seasonal affective disorder and their relevance to diurnal preference. Neuropsychopharmacology 28, 734–739. 10.1038/sj.npp.1300121, PMID: 12655319

[ref31] Jones K. H. Ellis J. Von Schantz M. Skene D. J. Dijk D. J. Archer S. N. (2007). Age-related change in the association between a polymorphism in the PER3 gene and preferred timing of sleep and waking activities. J. Sleep Res. 16, 12–16. 10.1111/j.1365-2869.2007.00561.x, PMID: 17309758 PMC7611878

[ref32] Kalmbach D. A. Arnedt J. T. Song P. X. Guille C. Sen S. (2017). Sleep disturbance and short sleep as risk factors for depression and perceived medical errors in first-year residents. Sleep 40:zsw073. 10.1093/sleep/zsw073, PMID: 28369654 PMC6084763

[ref33] Kim H. I. Lee H. J. Cho C. H. Kang S. G. Yoon H. K. Park Y. M.. (2015). Association of CLOCK, ARNTL, and NPAS2 gene polymorphisms and seasonal variations in mood and behavior. Chronobiol. Int. 32, 785–791. 10.3109/07420528.2015.1049613, PMID: 26134245

[ref34] Kim S. J. Lee J. H. Kim I. S. Jang K. H. Duffy J. F. (2012). Self-reported sleep duration, daytime sleepiness, and caffeine use in male and female morning and evening types. Sleep Med. Res. 3, 32–38. 10.17241/smr.2012.3.2.32

[ref35] Kitamura S. Hida A. Watanabe M. Enomoto M. Aritake-Okada S. Moriguchi Y.. (2010). Evening preference is related to the incidence of depressive states independent of sleep-wake conditions. Chronobiol. Int. 27, 1797–1812. 10.3109/07420528.2010.516705, PMID: 20969524

[ref36] Knapen S. E. Riemersma-van der Lek R. F. Antypa N. Meesters Y. Penninx B. W. Schoevers R. A. (2018). Social jetlag and depression status: results obtained from the Netherlands study of depression and anxiety. Chronobiol. Int. 35, 1–7. 10.1080/07420528.2017.1374966, PMID: 29111775

[ref37] Kryger M. H. Dement W. C. Roth T. (2010). Principles and practice of sleep medicine. 5th Edn. Philadelphia, PA: Saunders/Elsevier.

[ref38] Lavebratt C. Sjöholm L. K. Partonen T. Schalling M. Forsell Y. (2010a). PER2 variantion is associated with depression vulnerability. Am. J. Med. Genet. B Neuropsychiatr. Genet. 153, 570–581. 10.1002/ajmg.b.31021, PMID: 19693801

[ref39] Lavebratt C. Sjöholm L. K. Soronen P. Paunio T. Vawter M. P. Bunney W. E.. (2010b). CRY2 is associated with depression. PLoS One 5:e9407. 10.1371/journal.pone.0009407, PMID: 20195522 PMC2827563

[ref40] Lázár A. S. Slak A. Lo J. C. Y. Santhi N. von Schantz M. Archer S. N.. (2012). Sleep, diurnal preference, health, and psychological well-being: a prospective single-allelic-variation study. Chronobiol. Int. 29, 131–146. 10.3109/07420528.2011.641193, PMID: 22324552

[ref41] Lazzerini Ospri L. Prusky G. Hattar S. (2017). Mood, the circadian system, and melanopsin retinal ganglion cells. Annu. Rev. Neurosci. 40, 539–556. 10.1146/annurev-neuro-072116-031324, PMID: 28525301 PMC5654534

[ref42] Lee H. J. Rex K. M. Nievergelt C. M. Kelsoe J. R. Kripke D. F. (2011). Delayed sleep phase syndrome is related to seasonal affective disorder. J. Affect. Disord. 133, 573–579. 10.1016/j.jad.2011.04.046, PMID: 21601293 PMC3163003

[ref43] Lemoine P. Zawieja P. Ohayon M. M. (2013). Associations between morningness/eveningness and psychopathology: anepidemiological survey in three in-patient psychiatric clinics. J. Psychiatr. Res. 47, 1095–1098. 10.1016/j.jpsychires.2013.04.001, PMID: 23628386

[ref44] Levandovski R. Dantas G. Fernandes L. C. Caumo W. Torres I. Roenneberg T.. (2011). Depression scores associate with chronotype and social jetlag in a rural population. Chronobiol. Int. 28, 771–778. 10.3109/07420528.2011.602445, PMID: 21895489

[ref45] Li L. Wu C. Gan Y. Qu X. Lu Z. (2016). Insomnia and the risk of depression: a meta-analysis of prospective cohort studies. BMC Psychiatry 16:375. 10.1186/s12888-016-1075-3, PMID: 27816065 PMC5097837

[ref46] Liang Z. Martell M. A. C. (2018). Validity of consumer activity wristbands and wearable EEG for measuring overall sleep parameters and sleep structure in free-living conditions. J. Healthc. Inform. Res. 2, 152–178. 10.1007/s41666-018-0013-1 PMC8982823 35415400

[ref47] Liberman A. R. Halitjaha L. Ay A. Ingram K. K. (2018). Modeling strengthens molecular link between circadian polymorphisms and major mood disorders. J. Biol. Rhythm. 33, 318–336. 10.1177/0748730418764540, PMID: 29614896

[ref48] Liberman A. R. Kwon S. B. Vu H. T. Filipowicz A. Ay A. Ingram K. K. (2017). Circadian clock model supports molecular link between PER3 and human anxiety. Sci. Rep. 7, 1–10. 10.1038/s41598-017-07957-4, PMID: 28860482 PMC5579000

[ref49] Liu J. J. Hukic D. S. Forsell Y. Schalling M. Ösby U. Lavebratt C. (2015). Depression-associated ARNTL and PER2 genetic variants in psychotic disorders. Chronobiol. Int. 32, 579–584. 10.3109/07420528.2015.1012588, PMID: 25799324

[ref50] McCarthy M. J. Welsh D. K. (2012). Cellular circadian clocks in mood disorders. J. Biol. Rhythm. 27, 339–352. 10.1177/0748730412456367, PMID: 23010657

[ref51] McClung C. A. (2013). How might circadian rhythms control mood? Let me count the ways. Biol. Psychiatry 74, 242–249. 10.1016/j.biopsych.2013.02.019, PMID: 23558300 PMC3725187

[ref52] Mendlewicz J. (2009). Disruption of the circadian timing systems: molecular mechanisms in mood disorders. CNS Drugs 23, 15–26. 10.2165/11318630-000000000-00000, PMID: 19708722

[ref53] Merikanto I. Kronholm E. Peltonen M. Laatikainen T. Vartiainen E. Partonen T. (2015). Circadian preference links to depression in general adult population. J. Affect. Disord. 188, 143–148. 10.1016/j.jad.2015.08.061, PMID: 26363264

[ref54] Merikanto I. Lahti T. Kronholm E. Peltonen M. Laatikainen T. Vartiainen E.. (2013). Evening types are prone to depression. Chronobiol. Int. 30, 719–725. 10.3109/07420528.2013.784770, PMID: 23688117

[ref55] Moderie C. Van der Maren S. Paquet J. Dumont M. (2019). Home versus laboratory assessments of melatonin production and melatonin onset in young adults complaining of a delayed sleep schedule. J. Sleep Res. 29:e12905. 10.1111/jsr.12905, PMID: 31569275

[ref56] Mongrain V. Carrier J. Dumont M. (2006). Circadian and homeostatic sleep regulation in morningness-eveningness. J. Sleep Res. 15, 162–166. 10.1111/j.1365-2869.2006.00532.x, PMID: 16704571

[ref57] Nguyen C. Murray G. Anderson S. Filipowicz A. Ingram K. K. (2019). In vivo molecular chronotyping, circadian misalignment, and high rates of depression in young adults. J. Affect. Disord. 250, 425–431. 10.1016/j.jad.2019.03.050, PMID: 30878655

[ref58] Palagini L. Baglioni C. Ciapparelli A. Gemignani A. Riemann D. (2013). REM sleep dysregulation in depression: state of the art. Sleep Med. Rev. 17, 377–390. 10.1016/j.smrv.2012.11.001, PMID: 23391633

[ref59] Park Y. M. Seo Y. J. Matsumoto K. Shinkoda H. Park K. P. (1998). Sleep in relation to age, sex, and chronotype in Japanese workers. Percept. Mot. Skills 87, 199–215. 10.2466/pms.1998.87.1.199, PMID: 9760647

[ref60] Partonen T. Treutlein J. Alpman A. Frank J. Johansson C. Depner M.. (2007). Three circadian clock genes Per2, Arntl, and Npas2 contribute to winter depression. Ann. Med. 39, 229–238. 10.1080/07853890701278795, PMID: 17457720

[ref61] Pereira D. S. Tufik S. Louzada F. M. Benedito-Silva A. A. Lopez A. R. Lemos N. A.. (2005). Association of the length polymorphism in the human Per3 gene with the delayed sleep-phase syndrome: does latitude have an influence upon it? Sleep 28, 29–32. 10.3410/f.1024974.294734, PMID: 15700718

[ref62] Prat G. Adan A. (2013). Relationships among circadian typology, psychological symptoms, and sensation seeking. Chronobiol. Int. 30, 942–949. 10.3109/07420528.2013.790044, PMID: 23806000

[ref63] Preacher K. J. Hayes A. F. (2008). Asymptotic and resampling strategies for assessing and comparing indirect effects in multiple mediator models. Behav. Res. Methods 40, 879–891. 10.3758/brm.40.3.879 18697684

[ref64] Preacher K. J. Kelley K. (2011). Effect size measures for mediation models: quantitative strategies for communicating indirect effects. Psychol. Methods 16, 93–115. 10.1037/a0022658, PMID: 21500915

[ref65] Randler C. (2008). Morningness-eveningness, sleep-wake variables and big five personality factors. Personal. Individ. Differ. 45, 191–196. 10.1016/j.paid.2008.03.007

[ref66] Regestein Q. Natarajan V. Pavlova M. Kawasaki S. Gleason R. Koff E. (2010). Sleep debt and depression in female college students. Psychiatry Res. 176, 34–39. 10.1016/j.psychres.2008.11.006, PMID: 20079935

[ref67] Rique G. L. N. Fernandes Filho G. M. C. Ferreira A. D. C. De Sousa-Muñoz R. L. (2014). Relationship between chronotype and quality of sleep in medical students at the Federal University of Paraiba, Brazil. Sleep Sci 7, 96–102. 10.1016/j.slsci.2014.09.004, PMID: 26483910 PMC4521651

[ref68] Robillard R. Naismith S. Rogers N. L. Ip T. K. Hermens D. F. Scott E.. (2013). Delayed sleep phase in young people with unipolar or bipolar affective disorders. J. Affect. Disord. 145, 260–263. 10.1016/j.jad.2012.06.006, PMID: 22877966

[ref92] Roenneberg T. Kuehnle T. Juda M. Kantermann T. Allebrandt K. Gordijn M. (2007). Epidemiology of the human circadian clock. Sleep Med. Rev. 11, 429–438. 10.1016/j.smrv.2007.07.005 17936039

[ref69] Roepke S. E. Duffy J. F. (2010). Differential impact of chronotype on weekday and weekend sleep timing and duration. Nat. Sci. Sleep 2, 213–220. 10.2147/NSS.S12572, PMID: 20890372 PMC2947028

[ref70] Roeser K. Schlarb A. A. Kübler A. (2013). The chronotype-academic performance model (CAM): daytime sleepiness and learning motivation link chronotype and school performance in adolescents. Personal. Individ. Differ. 54, 836–840. 10.1016/j.paid.2012.12.021

[ref71] Sandman N. Valli K. Kronholm E. Revonsuo A. Laatikainen T. Paunio T. (2015). Nightmares: risk factors among the finnish general adult population. Sleep 38, 507–514. 10.5665/sleep.4560, PMID: 25325474 PMC4355890

[ref93] Shi S. Q. White M. J. Borsetti H. M. Pendergast J. S. Hida A. Ciarleglio C. M. (2016). Molecular analyses of circadian gene variants reveal sex-dependent links between depression and clocks. Transl. Psychiatry 6:e748. 10.1038/tp.2016.9 26926884 PMC4872462

[ref72] Simor P. Zavecz Z. Pálosi V. Török C. Köteles F. (2014). The influence of sleep complaints on the association between chronotype and negative emotionality in young adults. Chronobiol. Int. 32, 1–10. 10.3109/07420528.2014.935786, PMID: 25003651

[ref73] Sivertsen B. Harvey A. G. Pallesen S. Hysing M. (2015). Mental health problems in adolescents with delayed sleep phase: results from a large population-based study in Norway. J. Sleep Res. 24, 11–18. 10.1111/jsr.12254, PMID: 25358244

[ref74] Soehner A. M. Kennedy K. S. Monk T. H. (2011). Circadian preference and sleep-wake regularity: associations with self-report sleep parameters in daytime-working adults. Chronobiol. Int. 28, 802–809. 10.3109/07420528.2011.613137, PMID: 22080786 PMC4143130

[ref75] Soria V. Martínez-Amorós E. Escaramís G. Valero J. Pérez-Egea R. García C.. (2010). Differential association of circadian genes with mood disorders: CRY1 and NPAS2 are associated with unipolar major depression and clock and VIP with bipolar disorder. Neuropsychopharmacology 35, 1279–1289. 10.1038/npp.2009.230, PMID: 20072116 PMC3055337

[ref76] Steel Z. Marnane C. Iranpour C. Chey T. Jackson J. W. Patel V.. (2014). The global prevalence of common mental disorders: a systematic review and meta-analysis 1980-2013. Int. J. Epidemiol. 43, 476–493. 10.1093/ije/dyu038, PMID: 24648481 PMC3997379

[ref77] Turco M. Biscontin A. Corrias M. Caccin L. Bano M. Chiaromanni F.. (2017). Diurnal preference, mood and the response to morning light in relation to polymorphisms in the human clock gene PER3. Sci. Rep. 7, 1–10. 10.1038/s41598-017-06769-w, PMID: 28761043 PMC5537342

[ref78] Van den Berg J. F. Kivelä L. Antypa N. (2018). Chronotype and depressive symptoms in students: an investigation of possible mechanisms. Chronobiol. Int. 35, 1248–1261. 10.1080/07420528.2018.1470531, PMID: 29764217

[ref79] Van der Maren S. Moderie C. Duclos C. Paquet J. Daneault V. Dumont M. (2018). Daily profiles of light exposure and evening use of light-emitting devices in young adults complaining of a delayed sleep schedule. J. Biol. Rhythm. 33, 192–202. 10.1177/0748730418757007, PMID: 29463186

[ref80] Viola A. U. Archer S. N. James L. M. Groeger J. A. Lo J. C. Y. Skene D. J.. (2007). PER3 polymorphism predicts sleep structure and waking performance. Curr. Biol. 17, 613–618. 10.1016/j.cub.2007.01.073, PMID: 17346965

[ref81] Viola A. U. Tobaldini E. Chellappa S. L. Casali K. R. Porta A. Montano N. (2011). Short-term complexity of cardiac autonomic control during sleep: REM as a potential risk factor for cardiovascular system in aging. PLoS One 6:e19002. 10.1371/journal.pone.0019002, PMID: 21544202 PMC3081328

[ref82] Watson N. F. Harden K. P. Buchwald D. Vitiello M. V. Pack A. I. Strachan E.. (2014). Sleep duration and depressive symptoms: a gene-environment interaction. Sleep 37, 351–358. 10.5665/sleep.3412, PMID: 24497663 PMC3900629

[ref83] Wittmann M. Dinich J. Merrow M. Roenneberg T. (2006). Social jetlag: misalignment of biological and social time. Chronobiol. Int. 23, 497–509. 10.1080/07420520500545979, PMID: 16687322

[ref84] Wittmann M. Paulus M. Roenneberg T. (2010). Decreased psychological well-being in late ‘chronotypes’ is mediated by smoking and alcohol consumption. Subst. Use Misuse 45, 15–30. 10.3109/10826080903498952, PMID: 20025436

[ref85] Yadav A. Rani S. Singh S. (2016). Working ‘out-of-Phase’ with reference to chronotype compromises sleep quality in police officers. Chronobiol. Int. 33, 151–160. 10.3109/07420528.2015.1121876, PMID: 26785837

[ref86] Yadav A. Singh S. (2014). Relationship of chronotype to sleep pattern in a cohort of college students during work days and vacation days. Indian J. Exp. Biol. 52, 569–574. PMID: 24851422

[ref87] Yu L. Buysse D. J. Germain A. Moul D. E. Stover A. Dodds N. E.. (2011). Development of short forms from the PROMIS™ sleep disturbance and sleep-related impairment item banks. Behav. Sleep Med. 10, 6–24. 10.1080/15402002.2012.636266, PMID: 22250775 PMC3261577

[ref88] Yun J. A. Ahn Y. S. Jeong K. S. Joo E. J. Choi K. S. (2015). The relationship between chronotype and sleep quality in korean firefighters. Clin. Psychopharmacol. Neurosci. 13, 201–208. 10.9758/cpn.2015.13.2.201, PMID: 26243849 PMC4540041

[ref89] Zhai L. Zhang H. Zhang D. (2015). Sleep duration and depression among adults: a meta-analysis of prospective studies. Depress. Anxiety 32, 664–670. 10.1002/da.22386, PMID: 26047492

[ref90] Zhang L. Hirano A. Hsu P. K. Jones C. R. Sakai N. Okuro M.. (2016). A PERIOD3 variant causes a circadian phenotype and is associated with a seasonal mood trait. Proc. Natl. Acad. Sci. U. S. A. 113, E1536–E1544. 10.1073/pnas.1600039113, PMID: 26903630 PMC4801303

